# Cancer cells same as zombies reprogram normal cells via the secreted microenvironment

**DOI:** 10.1371/journal.pone.0288003

**Published:** 2023-07-28

**Authors:** Shadi Rabiee, Elham Hoveizi, Mahmood Barati, Ali Salehzadeh, Mohammad Taghi Joghataei, Shima Tavakol

**Affiliations:** 1 Cellular and Molecular Research Center, Iran University of Medical Sciences, Tehran, Iran; 2 Department of Biology, Rasht Branch, Islamic Azad University, Rasht, Iran; 3 Department of Biology, Faculty of Science, Shahid Chamran University of Ahvaz, Ahvaz, Iran; 4 Stem Cells and Transgenic Technology Research Center (STTRC), Shahid Chamran University of Ahvaz, Ahvaz, Iran; 5 Department of Medical Biotechnology, Iran University of Medical Sciences, Tehran, Iran; 6 Department of Anatomy, Faculty of Medicine, Iran University of Medical Sciences, Tehran, Iran; The Hormel Institute (University of Minnesota), UNITED STATES

## Abstract

The cancer microenvironment plays a crucial role in promoting metastasis and malignancy even in normal cells. In the present study, the effect of acidic and conditioned media of cancer cells (MDA-MB-231), separately and in combination, was studied for the first time on the cell death mechanisms and DNA methylation of normal fibroblasts (NIH/3T3). Cell survival of conditioned media was rescued by the addition of acidic media to conditioned media, as shown by the results. Cell metabolic activity is deviated in a direction other than the Krebs cycle by acidic media The mitochondrial metabolic activity of all groups was enhanced over time, except for acidic media. Unlike the highest amount of ROS in conditioned media, its level decreased to the level of acidic media in the combination group. Furthermore, cells were deviated towards autophagy, rather than apoptosis, by the addition of acidic media to the conditioned media, unlike the conditioned media. Global DNA methylation analysis revealed significantly higher DNA hypomethylation in acidic media than in normal and combination media. Not only were cells treated with conditioned media rescued by acidic media, but also DNA hypomethylation and apoptosis in the combination group were decreased through epigenetic modifications. The acidic and conditioned media produced by cancer cells can remotely activate malignant signaling pathways, much like zombies, which can cause metabolic and epigenetic changes in normal cells.

## 1. Introduction

According to the World Health Organization’s 2020 report, cancer continues to be a significant global health issue, with 19,292,789 cases and 9,958,133 annual deaths. It is currently the second leading cause of death worldwide [1]. In 2014, for the first time, it was hypothesized by Tavakol that normal cells adjacent to cancer cells undergo failed reprogramming and are turned into cancer cells under the influence of acidic media secreted by cancer cells. Our earlier study found that acidic pH had an inductive effect on genetic and epigenetic modifications in these cells and that before pluripotency regulators are activated, the C-myc gene is activated and self-renewal and maintenance genes are more highly expressed than pluripotency genes. This suggests that cancer cells undergo incomplete or failed cell reprogramming, which could be one of the key factors contributing to their development [2]. Later, part of this hypothesis was confirmed by Som et al, who showed that normal fibroblasts upregulate OCT4, a major gene in reprogramming, upon exposure to acidic media [3].

The extracellular pH (pH_e_) of cancer cells is in the range of 6.3 to 7, which is considered acidic. There are several theories discussing the reasons for this phenomenon. One possible explanation is that carbonic anhydrase IX (CAIX), acting as a pH-stat, stabilizes the acidic pHe [4]. Another possible reason is altered cell metabolism, known as the Warburg effect, in which cancer cells preferentially use glycolysis to produce ATP due to decreased oxidative phosphorylation in mitochondria and insufficient vascular supply [5]. As a result, cancer cells secrete high levels of lactic acid, leading to an acidic extracellular environment in tumor tissues, even in the presence of sufficient oxygen supply [5, 6]. Metabolic reprogramming is, in fact, one of the critical events in cancer cells that impact the cell signaling cascade.

The acidic pHe levels of tumor tissues can lead to a multitude of negative outcomes, such as chemotherapy resistance and increased proliferation, invasion, progression, and metastasis of cancer cells [3, 7]. Additionally, the neighboring normal cells may undergo a faulty reprogramming process, epigenetic modifications, and RNA processing alterations, resulting in their conversion to cancerous cells [2, 8, 9]. Moreover, it increases genetic instability and DNA double-strand breaks by reducing DNA repair mechanisms and enhancing topoisomerase II activity and reactive oxygen species (ROS) production [10, 11]. Acidic pHe prohibits the function of cancer suppressor cells such as natural killer and T cells and increases the function of immunosuppressive cells such as regulatory T cells. In other words, the acidic microenvironment derived from cancer cells affects the balance of different immune cells, leading to the inhibition of cancer suppressor cells such as natural killer cells and the promotion of immunosuppressive cells such as regulatory T cells. This contributes to tumor immune escape and makes it more difficult for the immune system to eliminate cancer cells [12, 13]. Increased caspase activity is likely another mechanism by which acidic stress may induce cell apoptosis [14]. Some studies indicate that acidic pHe may trigger tumor progression, while others suggest that it may induce apoptosis of cancer cells [14, 15]. Notwithstanding, there are some reports regarding the induction of pro-oncogenes by apoptosis. Therefore, two theories might be said in one word [16, 17].

Cancer cells not only exert their effect through the acidic pHe, but also remotely influence other cancer cells and, as seen in our study with normal cells, through biomolecules secreted by cancer cells. In fact, cancer cells are more dangerous than zombies, as they can turn healthy cells into malignant cells from a distance. The conditioned media of cancer cells contains biomolecules secreted from cancer cells, including exosomes, apoptotic bodies, and other vesicles. However, metabolic reprogramming in cancer cells is primarily through exosomes [5]. In the face of starvation and acidosis, cancer cells release more exosomes into the microenvironment, leading to tumor progression, extracellular matrix (ECM) remodeling, and tumor microenvironment (TME) reprogramming. They trigger a cell signaling cascade leading to alterations in ECM and cell adhesion, as well as premetastatic niche formation [18].

Apoptosis and autophagy are two important cell death and cell survival mechanisms [16]. It has been accepted that acidosis stress induces autophagy in cancer cells [19]. Autophagy can regulate the expression of the BH3-only protein p53 upregulated modulator of apoptosis (PUMA), thereby affecting the apoptotic threshold of the cells and “priming” the cell for apoptosis induction in response to cytotoxic agents [20]. Effective chemotherapy requires adequate and correct knowledge of cancer biology about cell death and viability signaling pathways of cancer cells and normal cells adjacent to them. To target each stage of cancer, you should know about the hot-point of that cancer’s stage to target it. Therefore, it is necessary to know about cell death and survival mechanisms beyond every stage and the effect of cancer cells on normal cells and how they turn normal cells into cancer cells and progress to metastasis in patients. It is important to know which part of the conditioned media and acidic media are more involved in malignancy, metastasis, metabolic and epigenetic reprogramming of normal cells adjacent to cancer cells.

In line with the hypothesis that cancer cells can influence nearby normal cells and transform them into malignant cells [2], the present study investigated, for the first time, the effect of three different media on apoptosis, autophagy, and global DNA methylation of normal fibroblast cells (NIH/3T3). The media included conditioned media derived from aggressive cancer cells (MDA-MB 231), acidic medium (pH 6.7) separately, and a combination of acidic plus conditioned media. Fibroblasts, which are one of the most abundant cells in the normal niche and surrounding cancer cells, were selected as the normal cells to evaluate the effect of the tumor microenvironment on them. The findings of this study will contribute to the management of cancer therapies; however, further in-depth studies are necessary to explore the effect of acidic stress and its mechanism on cancer and normal cell fate.

## 2. Materials and methods

### 2.1. Cell culture

The normal mouse fibroblast cell line NIH/3T3 and the hormone-independent and highly aggressive mammalian breast carcinoma cell line MDA-MB 231 were obtained from the Pasteur Institute of Iran (Tehran, Iran). The cell lines were cultured in T75 flasks with Dulbecco’s modified Eagle’s medium (DMEM) (GIBCO) high glucose supplemented with 10% heat-inactivated fetal bovine serum (FBS) (GIBCO) and 1% penicillin/streptomycin (BioIDEA, Tehran, Iran) at 37°C in 5% CO2 and 95% humidity.

Four media were evaluated in this study, including normal (N) and acidic (A) groups based on the pH of their medium. To obtain the acidic medium, the pH was first adjusted to 7.4 for 24 hours, and then it was changed to 6.7 using MES buffer. The low-nutrition environment makes cells under stress and produces a medium similar to the low-nutrition environment of tumor tissue. The conditioned media of cancerous cells (C) was prepared by culturing MDA-MB 231 cells in DMEM high glucose supplemented with 1% FBS normal (pH = 7.4) at 37°C in 5% CO2 and 95% moisture for 48 hours. The fourth group was the conditioned media of cancerous cells adjusted to pH 6.7, designated as CA.

### 2.2. MTT assay as a marker of cell metabolic activity

To evaluate the effect of normal, acidic, conditioned, and conditioned plus acidic media on the cell metabolic activity of NIH/3T3 cells, an MTT assay was used. NIH/3T3 cells at a concentration of 1×10^4^ cells/well (third passage; logarithmic phase) were seeded in triplicate in a 96-well plate containing DMEM high glucose supplemented with 10% FBS (GIBCO) and 1% penicillin/streptomycin (BioIDEA, Tehran, Iran). After 24 hours, the media was completely removed, and the cells were cultured in normal, acidic, conditioned, and conditioned plus acidic media for 48 and 72 hours. Then, the media were removed, and 100 μl of MTT (Sigma, St. Louis, MI; 0.5 mg/ml) was added for 4 hours. Afterward, 100 μl of dimethyl sulfoxide (DMSO; Sigma) was added to each well plate to dissolve the hydrazine crystals for up to 20 minutes. Absorption was measured at 570 nm wavelength using a microplate reader (BioTek, Winooski, VT). The assay was performed in triplicate, and the values provided are the normalized mean ± SD of at least three independent experiments.

### 2.3. Lactate dehydrogenase (LDH) assay

The LDH assay is a sensitive method for estimating cell membrane damage and necrosis in some parts. NIH/3T3 cells (third passage) at a concentration of 1×10^4^ cells/well were seeded in triplicate in a 96-well plate containing DMEM high glucose supplemented with 10% FBS (GIBCO) and 1% penicillin/streptomycin (BioIDEA, Tehran, Iran). The medium was replaced with normal, acidic, conditioned, and conditioned plus acidic media for 48 and 72 hours. LDH release was evaluated using an LDH assay kit (Roche, Berlin, Germany). In brief, 100 μL/well of cell supernatant was transferred into parallel well plates and mixed with 100 μL/well of the reaction mixture for 30 minutes at 22°C. The absorbance was read using a microplate reader (BioTek, Winooski, VT) at a wavelength of 490 nm.

To assess intracellular LDH, 100 μL/well Triton X100 was added to cells up to 72 hours post-treatment. Then, the cell lysate was mixed with LDH assay reagents, and color intensity was measured using a microplate reader (BioTek, Winooski, VT) at a wavelength of 490 nm. The assay was performed in triplicate, and the values provided are the normalized (cell number) mean ±SD of at least three independent experiments.

### 2.4. Intracellular ROS kinetic analysis using DCFH‐DA

To study the impact of normal, acidic, conditioned, and conditioned plus acidic media on intracellular ROS production of NIH/3T3 cells, the cells were cultured in a 24-well plate at a concentration of 1× 10^5^ cells/well at 37°C in 5% CO2 and 95% humidity for 24 hours. Then, the medium was replaced with normal, acidic, conditioned, and conditioned plus acidic media for 6 and 72 hours. The fluorescent dye 2′,7′‐dichlorofluorescein diacetate (DCFH‐DA) at the final concentration of 10 μM was added to the wells, and the kinetics of intracellular ROS was measured using a fluorescence microplate reader for 45 minutes. The assay was performed in triplicate, and the values provided are the normalized mean ± SD of three independent experiments. All data were normalized against cell numbers.

Moreover, the images of ROS‐positive cells were captured using an inverted fluorescence microscope (Olympus AX‐800, Shinjuku, Tokyo, Japan) coupled with a digital photographic camera (Leica, DC200, Wetzlar, Germany) for 24 hours.

### 2.5. Annexin V-FITC/propidium iodide using apoptosis measurement

Annexin V and propidium iodide (PI) are vital stains to study apoptosis and necrosis. However, quadrant 3 is related to the other cell deaths. In brief, cells (3×10^5^ cells/well) were seeded in a 12-well plate for 24 h and then treated with normal, acidic, conditioned and conditioned plus acidic media for 48 and 72 h. Cells were stained in step with the manufacturer’s instructions. Annexin V-FITC (Promokine, Germany) and propidium iodide (PI) (Sigma–Aldrich) stains were added. Cell death was analyzed by a FACS Calibur flow cytometer (Becton Dickinson). The assay was performed in triplicate, and the values provided are the normalized mean ± SD of two independent experiments.

### 2.6. Autophagy measurement using acridine orange (AO) staining

The vital AO is a green fluorophore acidoropic dye that can be trapped in acidic organelles such as autolysosomes in a pH-dependent manner. When AO becomes protonated in acidic vesicles, it emits bright red fluorescence. The red- to- green fluorescence intensity ratio (R/GFIR) of AO was used to assess autophagy in NIH/3T3 cells as an accessible and reliable method. NIH/3T3 cells were seeded in triplicate in a 24-well plate at a concentration of 1×104 cells/well for 24 hours. Then, the medium was replaced with normal, acidic, conditioned, and conditioned plus acidic media for 48 and 72 hours. Cells were stained with 1 μg/ml AO (2.7 μM). R/GFIR were analyzed using a fluorescence microplate reader (BioTek, Winooski, VT) at an excitation wavelength of 488 nm and emission wavelengths of 530 (green) and 620 (red) nm. The assay was performed in triplicate, and the values provided are the normalized mean ± SD of three independent experiments. Microscopic images were obtained from an inverted fluorescence microscope (Olympus AX‐800, Shinjuku, Tokyo, Japan) coupled with a digital camera (Leica, DC200, Wetzlar, Germany) after 72 hours.

### 2.7. Gene analysis using a quantitative reverse transcription-polymerase chain reaction

LC3 and P62 genes were evaluated using quantitative reverse transcription-polymerase chain reaction (qRT-PCR) at the mRNA level. NIH/3T3 cells were treated with normal, acidic, conditioned, and conditioned plus acidic media for 72 hours. Total RNA was extracted using the RNX-Plus kit (Sinaclon, Iran), and DNAse 1 treatment was applied. Random hexamer and oligo dt-primed cDNA synthesis was carried out using a cDNA synthesis kit (Takara, Japan). cDNA was used for 45-cycle RT-PCR in a Rotor-Gene Q real-time analyzer (Corbett, Australia) using the EvaGreen master mix. Each reaction was repeated three times, and the relative fold change in gene expression was quantified using the DDCt method. The β-actin gene was selected as an internal control. The duplicate experiments were repeated three times, and the mean ± SD was calculated. The primer sequences of the genes were provided as follows.

β-Actin

F: 5´-TGAAGATCAAGATCATTGCTCCTC- 3´

R: 5´-TCAGTAACAGTCCGCCTAGAAG- 3´

P62

F: 5´-TGTGGAACATGGAGGGAAGAG- 3´

R: 5´-TGTGCCTGTGCTGGAACTTTC- 3´

LC3

F: 5´-GACGGCTTCCTGTACATGGTTT- 3´

R: 5´-TGGAGTCTTACACAGCCATTGC- 3´

### 2.8. Western blotting

Western blotting was used to evaluate the levels of Bax, Bcl2, P53, and LC3-II in NIH/3T3 cells. In brief, after treatment with normal, acidic, conditioned, and conditioned plus acidic media for 7 days, cells were prepared for protein extraction. Seven days was selected to evaluate the protein expression of apoptotic and autophagic proteins over a longer period. Proteinase A and RIPA buffer (25 mM Tris-Cl pH 7.6, 150 mM sodium chloride, 1% NP-40, 1% sodium deoxycholate, 1% sodium dodecyl sulfate, 1 mM phenylmethylsulfonyl fluoride, 1 mM sodium orthovanadate) were added to lyse the cells, and total protein concentrations in lysates were measured using a BCA protein assay kit. Protein separation was performed by SDS-PAGE, and then, the resolved proteins were transferred to PVDF membranes (Millipore, USA). After blocking for 2 hours at room temperature with 5% nonfat skim milk (DIFCO, Paris, France) in TBS-T [Tris-buffered saline containing 0.01% Tween 20 (Sigma Aldrich)], the membranes were incubated overnight at 4°C with the following primary antibodies: P53 (R&D SYSTEMS, CAT N Mab1746; 1:5000), LC3B (Cell Signaling, CAT N #3868; 1:1000), and β-actin (PADZA Padtan Pajooh, IRAN, CAT N MB106). The membranes were washed three times with TBS-T and further exposed to rabbit anti-mouse IgG-HRP conjugate (PADZA Padtan Pajooh, IRAN, 1;2000) and sheep anti-rabbit IgG-HRP conjugate (PADZA Padtan Pajooh, IRAN, 1;4000) as the secondary antibodies at room temperature for 1.5 h. Bands were clarified by enhanced chemiluminescence (Clarity™ western ECL The substrate, BioRad). Protein levels were normalized to beta-actin protein using ImageJ software (NIH, USA). The duplicate experiments were analyzed, and the mean ± SD was calculated.

### 2.9. DNA GC–MS

The method used in this study is considered a highly precise and authentic method for the determination of global DNA methylation. Gas chromatography-mass spectrophotometry (GC–MS) is a highly precise and authentic method for the determination of global DNA methylation. DNA methylation is one of the major epigenetic changes. In mammalian cells, carbon 5 in cytosine is the only site of DNA methylation. Various studies have shown an association between extensive DNA hypomethylation and various diseases, including cancer. Briefly, NIH/3T3 cells were treated with normal, acidic, conditioned and conditioned plus acidic media for 7 days. Seven days was selected to evaluate DNA methylation over a longer period. Then, DNA was extracted using the DNPTM Cinaclone kit. The quality and quantity of the extracted DNA were evaluated by a NanoDrop™ One^C^ (Thermo Scientific).

In the next step, chemical hydrolysis and derivatization of DNA were performed. First, 5 μg of genomic DNA was liquefied in 500 μL of 88% formic acid and then hydrolyzed at 140°C for 90 min in a glass vial. The derivatization was carried out after drying the hydrolysate by adding 25 μL of BSTFA (with 1% TMCS) plus 25 μL of acetonitrile at 30°C for 30 min; eventually, 2 μl of the derivatization mixture was injected into the GC–MS for analysis. GC–MS analysis was performed by Thermo GCMS-QP2000 (Thermo Finnigan San Jose, CA). Gas chromatographic separations were obtained on a DB5 capillary column (30 m * 0.25 mm * 0.25 m) with a film thickness (Phenomenex Torrance, CA) of 0.25 m.

### 2.10. Statistical analysis

The data were statistically analyzed using GraphPad Instat v3 software. Each triplicate experiment was repeated three times. The mean ± SD was used to express the results of the experiments. To compare the differences between various groups, one-way ANOVA was applied. Statistical significance was considered when the P value was less than 0.05.

## 3. Results

### 3.1. Metabolic activity assay using MTT assay

**Fig 1:**
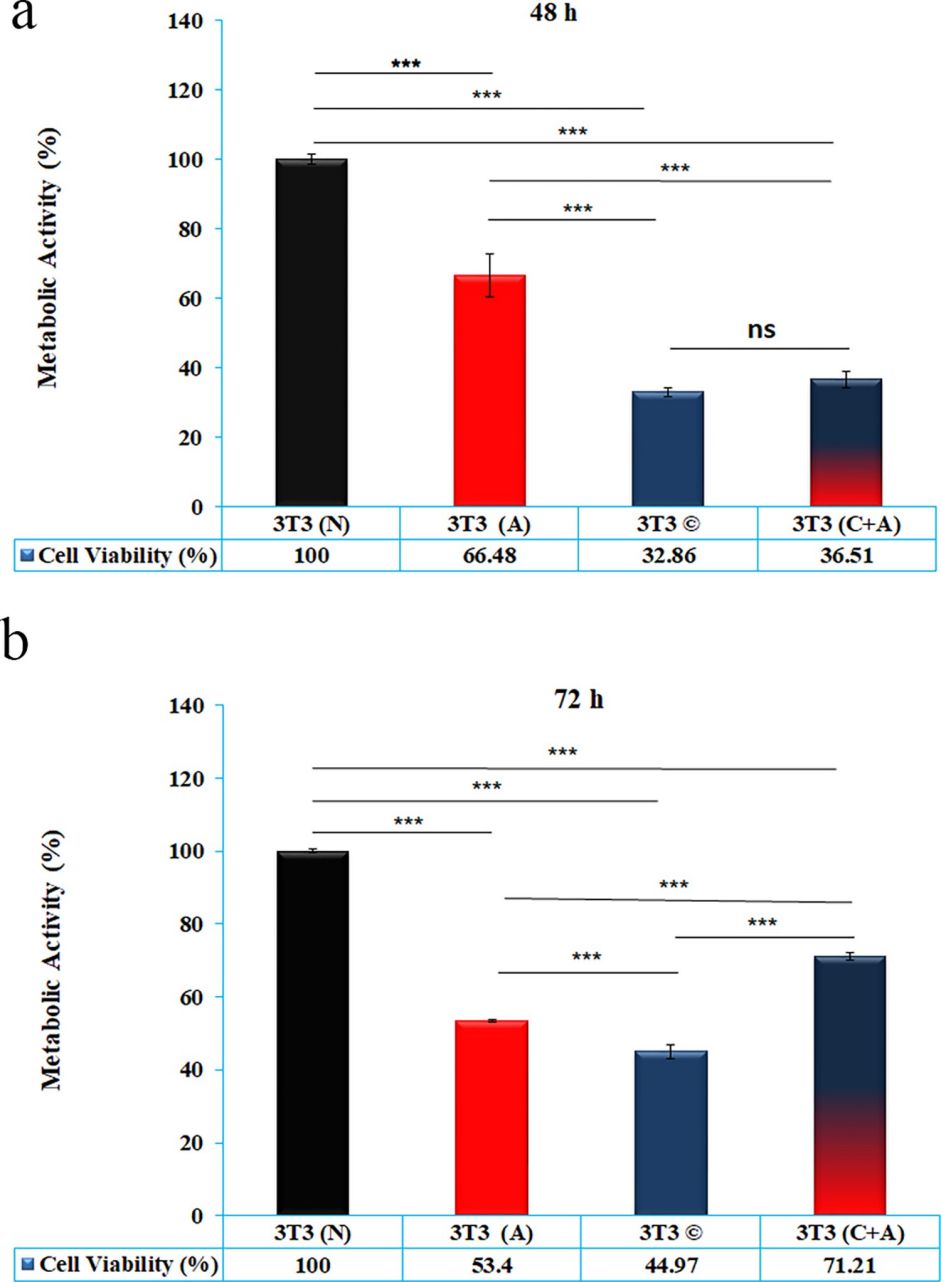
**a)** MTT assay of NIH/3T3 cells treated with normal media (N), acidic media (A), conditioned media (C) and acidic plus conditioned media (C + A) after 48 h. b) MTT assay of NIH/3T3 cells treated with normal media (N), acidic media (A), conditioned media (C) and acidic plus conditioned media (C + A) after 72 h. “ns” indicates p> 0.05 was considered nonsignificant. Other groups had significant differences in mitochondrial metabolic activity.

The MTT assay was performed to determine the effect of normal, acidic, conditioned and conditioned plus acidic media on mitochondrial succinate dehydrogenase activity of NIH/3T3 cells up to 48 and 72 h. The results showed less cell metabolic activity in all groups compared to the control group at 48 (P<0.001) and 72 h (P<0.001). Metabolic activity was significantly higher in acidic media than in conditioned and conditioned plus acidic media for 48 h (P<0.001). There was no significant difference between the cell metabolic activity of conditioned and conditioned plus acidic media 48 h posttreatment (P>0.05) (Fig 1A).

Although there was significantly less cell metabolic activity in cells treated with acidic media at 48 h compared to 72 h (p = 0.0054), conditioned (p< 0.0001) and conditioned plus acidic media (p< 0.0001) enhanced cell metabolic activity at 72 h compared to 48 h (Fig 1A and 1B). The data showed a significant decrease in cell metabolic activity in all groups compared to the control group at 72 h (p < 0.0001). The conditioned media induced significantly less cell metabolic activity than the acidic and conditioned plus acidic media. Although the cell metabolic activity of conditioned plus acidic media was less than that of the acidic media at 48 h, its cell metabolic activity was enhanced over time and was greater than that of acidic media at 72 h (p < 0.0001) (Fig 1B).

### 3.2. LDH release

**Fig 2:**
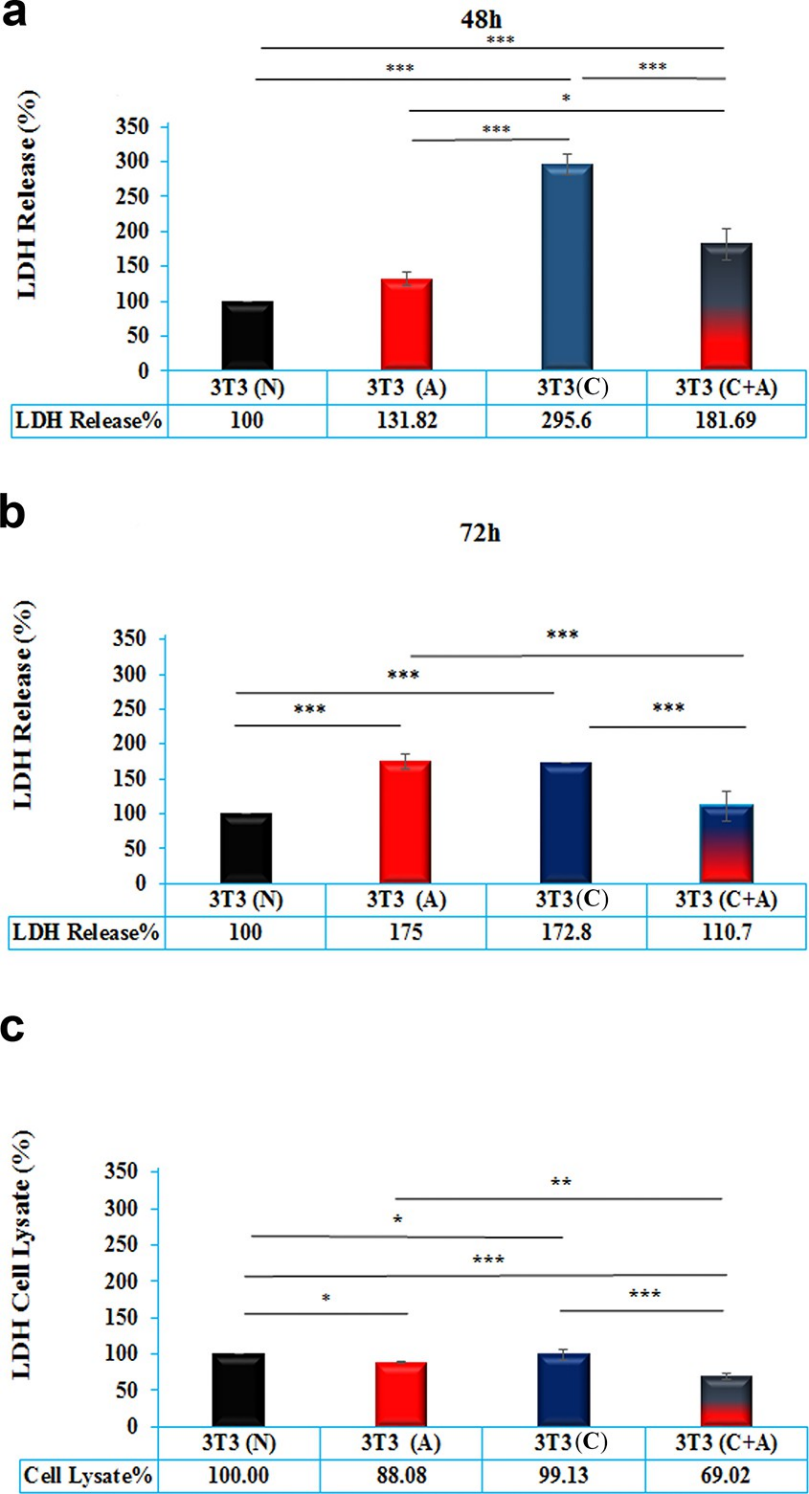
**a)** LDH release from NIH/3T3 cells treated with normal media (N), acidic media (A), conditioned media (C) and acidic plus conditioned media (C + A) after 48 h. b) LDH release from NIH/3T3 cells treated with normal media (N), acidic media (A), conditioned media (C) and acidic plus conditioned media (C + A) after 72 h. c) Cytoplasmic LDH content of NIH/3T3 cells treated with normal media (N), acidic media (A), conditioned media (C) and acidic plus conditioned media (C + A) after 72 h. * indicates p<0.05, ** indicates p<0.01 and *** indicates p< 0.001.

Cell membrane damage was evaluated using LDH release at 48 h. Data were normalized to cell number at 48 h. The results showed significantly higher LDH release from the treated cells compared to the control group (p < 0.001). Conditioned media induced significantly higher LDH release compared to the acidic and conditioned plus acidic media, while the LDH release of conditioned plus acidic media was higher than acidic media (p < 0.001) (Fig 2A).

LDH release results at 72 h showed that acidic and conditioned media induced significantly higher LDH release compared to the normal and condition plus acidic media (P< 0.001), while there was no significant difference between the LDH release from the control and conditioned plus acidic media (P> 0.05). In addition, there was no significant difference between LDH release from the acidic and conditioned media (P> 0.05) (Fig 2B).

**Fig 3:**
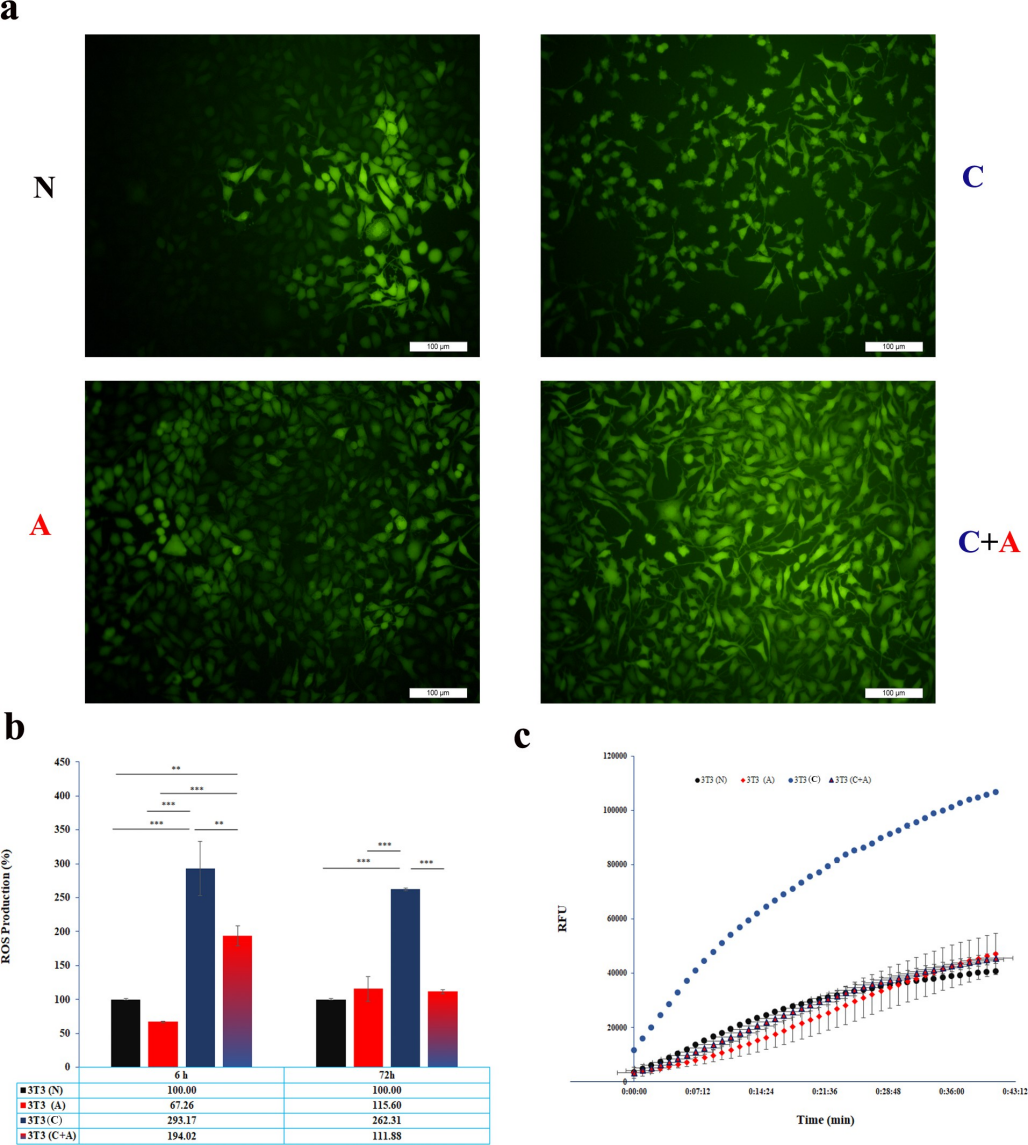
**a)** Intracellular ROS staining of NIH/3T3 cells treated with normal media (N), acidic media (A), conditioned media (C) and acidic plus conditioned media (C + A) after 24 h. **b)** The percentage of intracellular ROS production from NIH/3T3 cells treated with normal media (N), acidic media (A), conditioned media (C) and acidic plus conditioned media (C + A) after 6 and 72 h. **c**) The kinetics of intracellular ROS production in NIH/3T3 cells treated with normal media (N), acidic media (A), conditioned media (C) and acidic plus conditioned media (C + A) after 72 h. Data were normalized against cell number. * indicates p<0.05, ** indicates p<0.01 and *** indicates p< 0.001.

Moreover, the cytoplasmic LDH concentration in treated cells showed that acidic (P< 0.05) and conditioned plus acidic media (P< 0.001) induced less LDH production in cells compared to the normal and conditioned media. However, there was no significant difference between the amount of LDH in cells treated with normal and conditioned media (P> 0.05). Conditioned plus acidic media induced significantly less LDH production in cells than acidic media (P< 0.01) (Fig 3B).

### 3.3. Intracellular ROS kinetic analysis using DCFH-DA

An index of oxidative stress in cells, intracellular ROS kinetics, was investigated after 24 h (Fig 3A). It was found that significantly higher intracellular ROS production was induced by the conditioned media compared to the normal (P<0.001), acidic (P<0.001), and conditioned media plus acidic groups (P<0.01). Although the addition of conditioned media to acidic media decreased intracellular ROS production compared to the conditioned group (P<0.01), its intracellular ROS production level was still higher than that of the normal (P<0.01) and acidic groups (P<0.001). In other words, the level of intracellular ROS production was significantly decreased in acidic media compared with other groups at 6 h. The level of intracellular ROS production in the conditioned media was 4.36 times higher than that in the acidic media, while when it was added to acidic media, its level was decreased to 2.88 (Fig 3B).

Although the level of intracellular ROS production in the acidic group was enhanced by 72 h compared to 6 h (1.72 times), its level was significantly decreased in the conditioned plus acidic group at 72 h compared to 6 h (0.58 times). The results derived from conditioned media showed that as before, the level of intracellular ROS production in conditioned media was higher than that in the other groups (P<0.001), while there was no significant difference between the acidic and normal groups (p>0.05). Moreover, there was no significant difference in intracellular ROS production between the acidic and conditioned plus acidic groups (p>0.05) (Fig 3B and 3C).

### 3.4. Apoptosis measurement by Annexin V-FITC/PI

**Fig 4:**
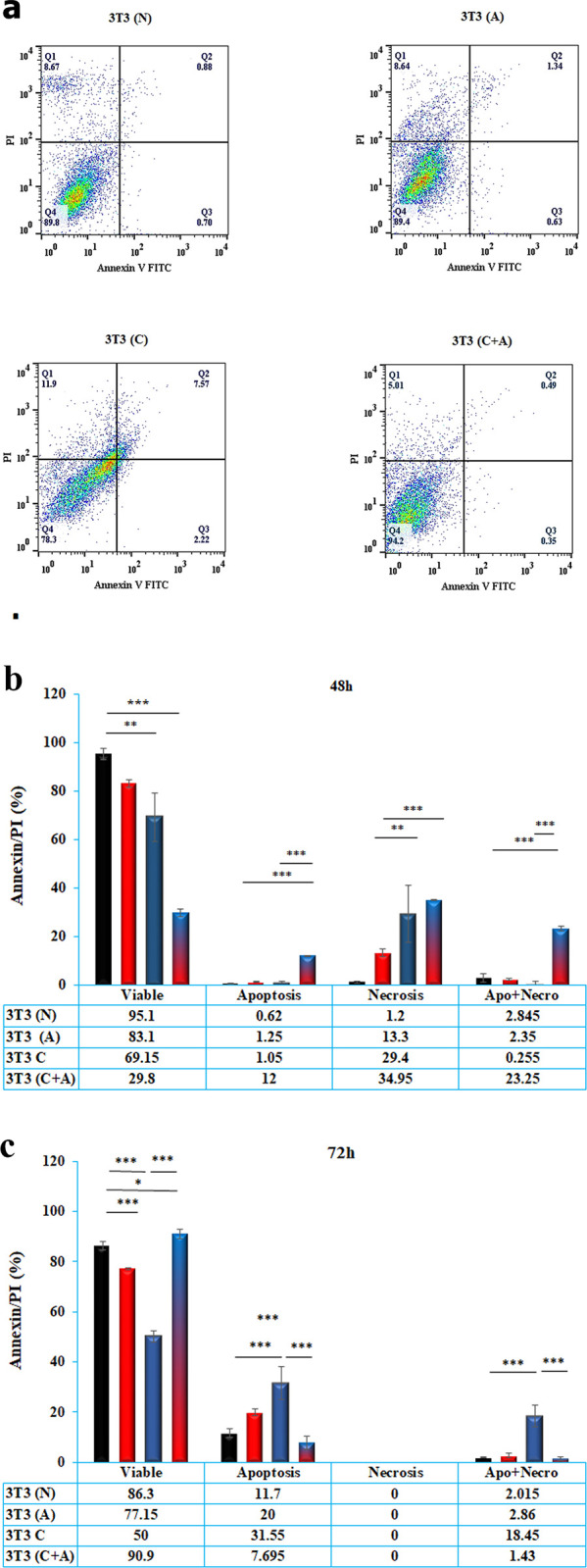
**a)** Annexin/PI graphs of NIH/3T3 cells treated with normal media (N), acidic media (A), conditioned media (C) and acidic plus conditioned media (C + A) after 48 h. Early apoptosis is shown in the bottom right quadrant (Q3) for each panel, while late apoptosis (Annexin+/PI+) is shown in the upper right quadrant (Q2). Viable cells are represented in the lower left quadrant (Q4). Necrosis (Annexin −/PI+) is shown in the upper left quadrant (Q1). **b)** Annexin/PI staining data from NIH/3T3 cells treated with normal media (N), acidic media (A), conditioned media (C) and acidic plus conditioned media (C + A) after 48 h. Viability was decreased and necrosis was increased in A, C, and C+A media compared to N media. Apoptosis-necrosis and necrosis were significantly observed in C+A cells compared to other cells. **c)** Annexin/PI staining data from NIH/3T3 cells treated with normal media (N), acidic media (A), conditioned media (C) and acidic plus conditioned media (C + A) after 72 h. * indicates p value <0.05, ** indicates p value <0.01 and *** indicates p value < 0.001.

Annexin/PI staining was used to evaluate the cell death mechanisms related to early apoptosis, necrosis, late apoptosis and other unknown cell deaths [21]. As depicted in Fig 4A and 4B, the data showed that conditioned plus acidic media induced significantly higher numbers of early and late apoptotic cells (P< 0.001) than the other treatments. There was no significant difference between necrotic cells derived from conditioned and conditioned plus acidic cells (P> 0.05). There was no significant difference between the number of early and late apoptotic cells in normal, acidic and conditioned media (P> 0.05), while the number of necrotic cells derived from conditioned media was significantly higher than that derived from acidic (P< 0.05) and normal (P< 0.01) media by 48 h (Fig 4B).

The number of viable cells treated with conditioned plus acidic media was found to be higher than that in the other groups after 72 h. Moreover, the number of apoptotic cells treated with conditioned plus acidic media was significantly lower than that in the acidic (P< 0.05) and conditioned media groups (P< 0.001). In other words, the conditioned media induced significantly higher early apoptosis in cells compared to the other groups (P< 0.001). Additionally, the conditioned media induced significantly higher late apoptosis and eventually other cell death types in cells compared to the other groups (P< 0.001). However, there was no significant difference between the number of late apoptotic cells in normal, acidic, and conditioned plus acidic cells (P> 0.05) (Fig 4C).

### 3.5. Autophagy measurement by AO staining

**Fig 5:**
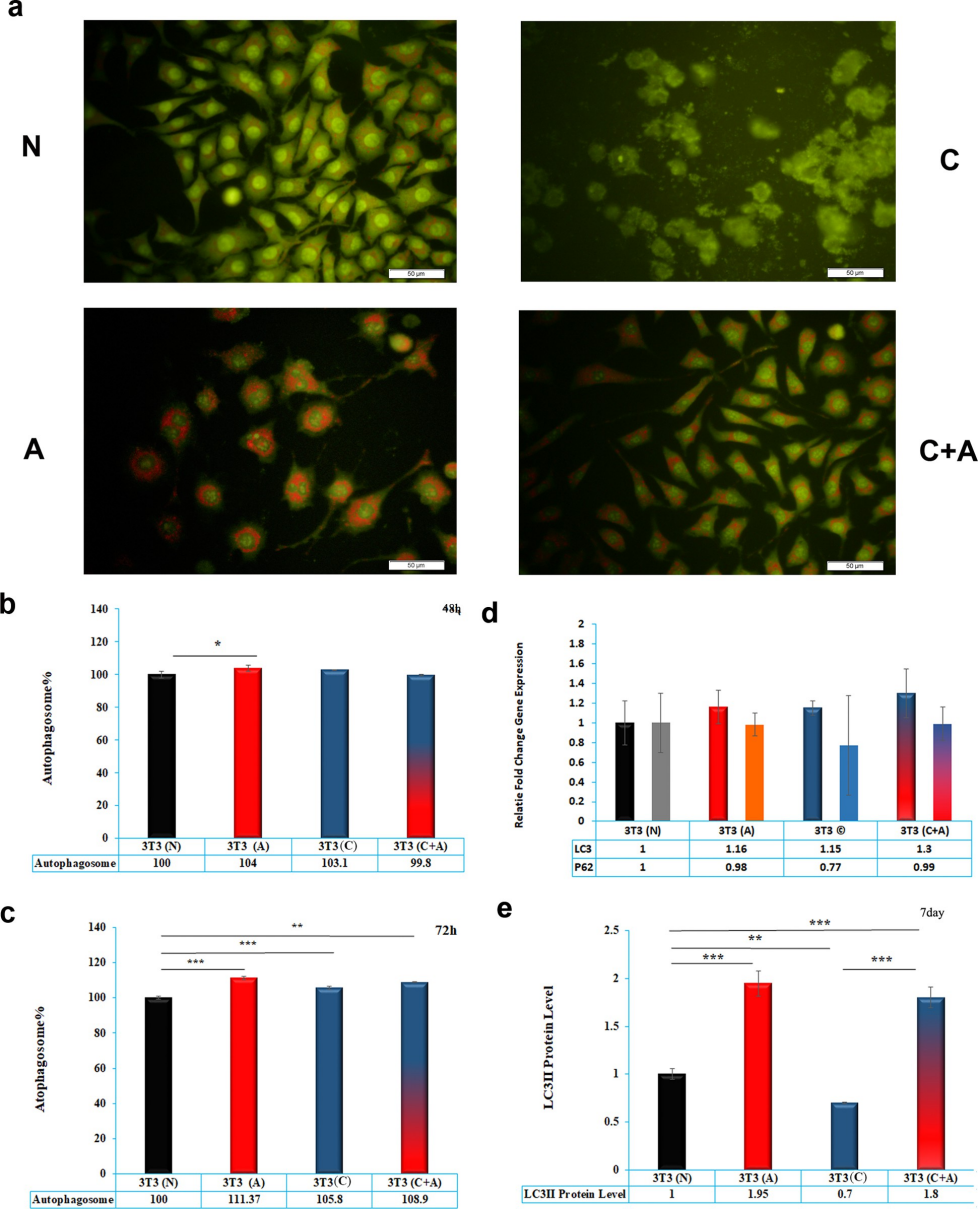
**a**) Acridine orange staining of NIH/3T3 cells treated with normal media (N), acidic media (A), conditioned media (C) and acidic plus conditioned media (C + A) after 72 h. **b**) Autophagosome percentage in NIH/3T3 cells treated with normal media (N), acidic media (A), conditioned media (C) and acidic plus conditioned media (C + A) after 48 h. **c**) Autophagosome percentage in NIH/3T3 cells treated with normal media (N), acidic media (A), conditioned media (C) and acidic plus conditioned media (C + A) after 72 h. **d)** Real-time PCR data related to p62 and LC3 gene analysis of NIH/3T3 cells treated with normal media (N), acidic media (A), conditioned media (C) and acidic plus conditioned media (C + A) after 72 h. **e)** Western blotting of LC3-II/I protein of NIH/3T3 cells treated with normal media (N), acidic media (A), conditioned media (C) and acidic plus conditioned media (C + A) after 7 days. * indicates p<0.05, ** indicates p<0.01 and *** indicates p< 0.001.

AO staining or the R/G ratio is a valuable index to measure autophagy in cells. The results indicated that acidic media significantly induced autophagy in cells compared to the normal and conditioned plus acidic media (P< 0.001) by 48 h, while there was no significant difference between the percentage of autophagy in acidic and conditioned media (P> 0.05) (Fig 5B). However, by the time and after 72 h, all groups induced significantly higher autophagy compared to the normal group (P< 0.001) (Fig 5A and 5C). Conditioned media induced significantly fewer autophagy vacuoles in cells compared to the acidic and conditioned plus acidic media (P< 0.001).

### 3.6. Gene analysis

To evaluate the expression of P62 and LC3 genes in cells treated with acidic, conditioned, and acidic plus conditioned media, real-time PCR was performed. The results showed that there was no significant difference in the fold change gene expression of P62 and LC3 in all groups (p>0.05) (Fig 5D).

### 3.7. Western blotting

Western blotting was used to evaluate the levels of proteins involved in cell death, including Bax, Bcl2, P53 and LC3-II, for 7 days. LC3-II as an index of autophagy showed that acidic media induced significantly higher LC3-II protein compared to the normal and conditioned media (P< 0.0001), while there was no significant difference between acidic and conditioned plus acidic media (P> 0.05). Furthermore, conditioned media induced significantly less LC3-II protein compared to the other treatments (P< 0.0001) (Fig 5E).

**Fig 6:**
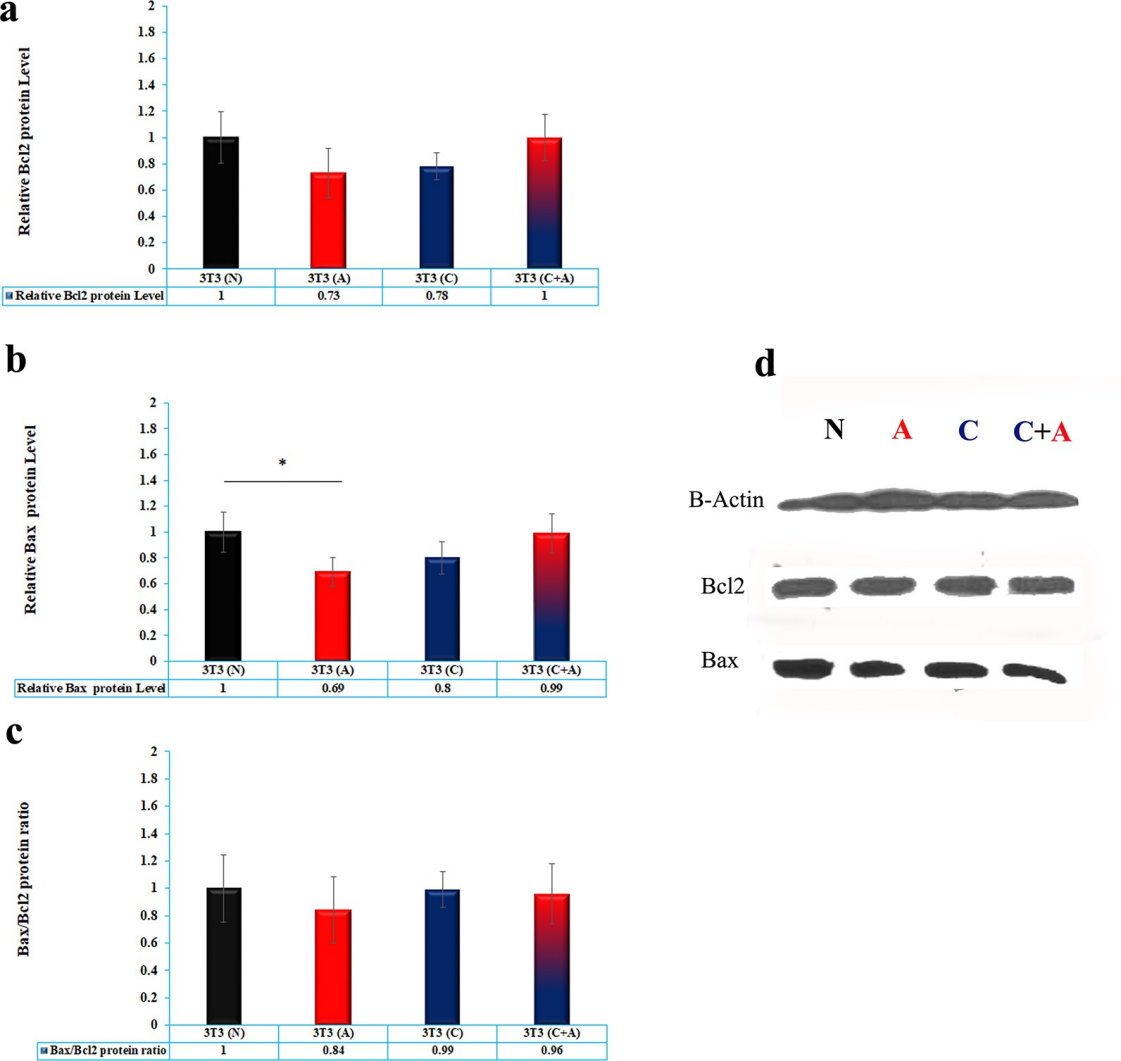
**a)** Western blotting of Bcl2 protein. b) Bax. c) Bax/bcl2 ratio. d) Figures of beta-actin, bax and Bcl2 in NIH/3T3 cells treated with normal media (N), acidic media (A), conditioned media (C) and acidic plus conditioned media (C + A) after 7 days. * Mean p<0.05.

The results showed that although there was no significant difference between the level of Bcl2 and the ratio of Bax/Bcl2 (P> 0.05), the level of Bax protein in acidic media was significantly less than that in normal media (p = 0.0491). There was no significant difference in the level of Bax protein in cells treated with conditioned, normal and conditioned plus acidic media (Fig 6A-6D).

### 3.8. DNA GC–MS

**Fig 7:**
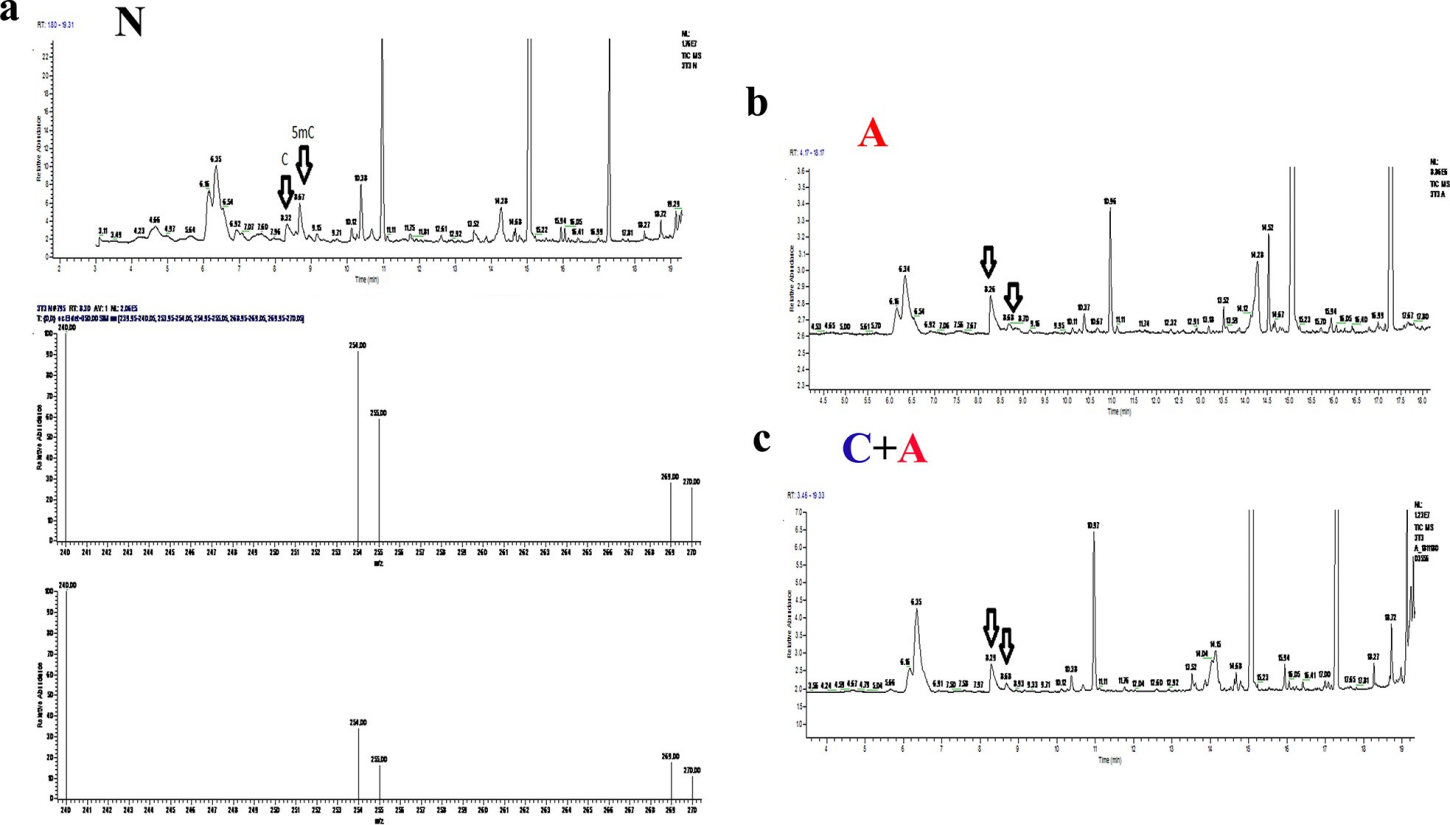
DNA gas chromatography-mass spectrophotometry (GC–MS) to detect DNA methylation in CpG islands of a) NIH/3T3 cells treated with normal media (N), b) acidic media (A) and C) acidic plus conditioned media (C + A) at 7 days post-treatment.

To evaluate the quantity of DNA methylation in normal cells in the face of acidic media and acidic plus conditioned media, DNA GC–MS was performed. The DNA GC–MS data on 5 methylcytosines showed that acidic media decreased global DNA methylation. Specifically, the percentage of 5 methylcytosines in normal cells (62.41± 1.13%) was significantly higher than that in cells treated with acidic media (15.67±0.46%) (P value = 0.001). On the other hand, the addition of acidic media plus conditioned media to normal cells (22.34± 0.6) increased DNA methylation compared to the acidic and normal media (P value = 0.001) (Fig 7). However, it was not possible to measure the content of DNA methylation in conditioned media.

## 4. Discussion

The microenvironment of cancer is generally acidic compared to normal cells, and previous studies have shown that an acidic extracellular pH can affect the expression of various genes in cells [22]. In this study, we evaluated the effect of acidic medium (pH 6.7), conditioned media obtained from MDA-MB-231 cells, and acidic plus conditioned media on apoptosis and autophagy in normal fibroblast cells. The MTT assay data indicated that the mitochondrial succinate dehydrogenase activity of normal fibroblasts was decreased in the presence of acidic, conditioned media, and conditioned plus acidic media. However, the acidic media rescued cell metabolic activity when cells were treated with conditioned media. The trend of LDH release data was consistent with the MTT assay data. Although there was no significant difference between the Bax/Bcl2 protein ratio of groups, Annexin-PI flow cytometry data showed that conditioned plus acidic media induced significantly higher early apoptosis than the other groups at 48 h, but the scenario changed at 72 h, and conditioned media induced significantly higher cell apoptosis. Moreover, acidic and conditioned plus acidic media did not induce significant apoptosis compared to normal media and instead induced autophagy. Furthermore, DNA hypomethylation was observed in all groups compared to normal media, but it decreased in the combination group compared to acidic media.

The MTT assay is not only a cell viability analysis but also a metabolic assay that measures mitochondrial succinate dehydrogenase. Therefore, in cancer cells that may alter the Krebs cycle, the MTT assay is more of a metabolic assay than cell viability [2]. In the present study, cell viability was checked by Annexin-PI flowcytometry and compared to MTT assay data as a metabolic assay. MTT assay data showed that conditioned media significantly decreased mitochondrial succinate dehydrogenase activity compared to others; however, the activity increased from 48 to 72 h. Moreover, the mitochondrial succinate dehydrogenase activity of conditioned plus acidic media was higher than conditioned and less than acidic media by 48 h. Enzyme or metabolic activity of the combination group significantly increased compared to the acidic media by 72 h. It seems the combination can enhance metabolic activity and alter the cell signaling cascade toward the Krebs cycle. However, the metabolic activity of acidic media was decreased from 48 to 72 h, while it was still higher than conditioned media. In other words, the metabolic activity of all groups was enhanced over time except for acidic media. Cell metabolic activity and cell viability data were derived from the MTT assay and Annexin-PI staining, respectively. Live cell data showed that the percentage of live cells in conditioned media was higher than the succinate dehydrogenase activity at 48 h. In other words, conditioned media provoked cells to choose another metabolic pathway in addition to Krebs. However, over time, the difference between viable cells and Krebs enzyme activity was not large. Since the number of live cells by flowcytometry was 50% and succinate dehydrogenase activity was 45%, it is not clear that approximately 55% of the remaining cells chose glycolysis and glutamine mechanisms instead of Krebs. On the other side, it might be said that most live cells that are not undergoing cell death mechanisms, such as early and late apoptosis and autophagy, etc. completely chose the Krebs cycle at 72 h, while cells under cell death mechanisms selected glycolysis and glutamine mechanisms. These ambiguities are mysterious, and revealing this secret will be a great help in cancer biology. Anyway, the activity of succinate dehydrogenase, an index of the Krebs cycle, decreased to 45%. These data were in accordance with Zhao et al. [5]. They demonstrated that exosomes derived from cancer-associated fibroblasts (CAFs) suppress mitochondrial oxidative phosphorylation and instead enhance glycolysis and glutamine-dependent reductive carboxylation.

Interestingly, over time, the number of cells treated with conditioned plus acidic media significantly increased, and most of the metabolic activity belonged to the Krebs cycle. It seems that other metabolic revolutions, perhaps glycolysis or glutamine-dependent reductive carboxylation, were added. There was an approximately 20% difference between viable cells (~90%) and Krebs enzyme activity (~71%) in conditioned plus acidic media at 72 h, while at 48 h, the difference was not as great. To more deeply discuss, the trend of metabolic activity in acidic media would be valuable. There was a difference of 17–24% between live cells and Krebs enzyme activity at 48 and 72 h. However, the activity of succinate dehydrogenase dramatically decreased in acidic media, unlike conditioned media. In summary, it seems that acidic media diverts cells in a direction other than the Krebs cycle, and by the time its effect decreases, the cell metabolic activity of conditioned plus acidic media increases.

Cancer cells export extracellular vesicles, such as exosomes and apoptotic bodies, involved in metastasis, stemness, angiogenesis, etc. Therefore, cancer-conditioned media that are a part of the TME and the mirror of donor cells, cancer cells, play a critical role in tumor progression and ECM remodeling. In other words, both mutation and the microenvironment play key roles in tumor initiation. In some cases, the elimination of exosomes suppresses tumor progression [18]. It was demonstrated that the addition of exosomes derived from cancer cells enhances glucose uptake and LDH release as two indexes of glycolysis [5]. In addition, LDH is a key biomarker of cell membrane function. It is rapidly released into the cell microenvironment when the plasma membrane is damaged during apoptosis, necrosis, or other forms of cellular damage [23–26].

The trend of LDH release data was in good agreement with MTT assay data. The first point is that all groups induced significantly higher LDH release than normal media, while the intracellular content of LDH was less than that in normal media in all groups except for conditioned media. The intracellular LDH content of acidic media was higher than that of conditioned plus acidic media. It is accepted that hypoxia upregulates HIF-1 and, in turn, upregulates LDHA. However, in the present study, it was shown that despite the absence of hypoxia, intracellular LDH increased. This might be due to the fact that cancer cells upregulating carbonic anhydrase IX (CAIX) as a pHe-stat have higher intracellular LDH than normal cells [4].

There was no significant difference between acidic and conditioned media to induce intracellular LDH content and LDH release at 72 h. On the other hand, conditioned media induced significantly higher LDH release than other groups, and its level was significantly decreased from 48 to 72 h. The addition of acidic to conditioned media decreased LDH release compared to conditioned media, and over time its level decreased even less than acidic media. The LDH release level in cells treated with acidic media was less than in conditioned and combination media groups at 48 h. Despite other groups that LDH release decreased over time, its level increased by the time from 48 to 72 h. Collectively, it might be said based on MTT assay, Annexin-PI data and LDH release, the addition of acidic media rescues cell survival of conditioned media and by the time acidic media deviates cell metabolic activity toward higher LDH release may be through glycolysis mechanisms. It is not clear when LDH release derived from acidic media has increased over time, so how does it in addition to conditioned media decreases LDH release. It would be beneficial to receive clarification on whether or not these changes are a result of the modulation of metabolic mechanisms such as the Krebs cycle, glycolysis, glutamine-dependent reductive carboxylation, and others.

To further explore the cell behavior in the face of the conditioned and acidic media, iROS production was measured. iROS and subsequent oxidative stress may be another reason for the decreased cell viability in conditioned media. The trend was the same as the LDH release data, in which, except for acidic media, iROS drastically decreased from 6 to 72 h. The amount of iROS was higher than others in conditioned media, while when acidic media was added to conditioned media, its level was dramatically decreased to the level of acidic media. ROS interact with lipids in the cell membrane, resulting in LDH release from the cytoplasm to the microenvironment [27, 28]. Based on the ROS data, we expected that LDH release from the acidic media would decrease. Therefore, it seems that some mechanisms result in LDH release enhancement. It is not clear whether acidic media damage the cell membrane in a ROS-independent mechanism or whether LDH release is a byproduct of anaerobic metabolism. Notwithstanding the cytoplasmic LDH and lower level of intracellular LDH compared to normal cells in acidic media, it seems that cell membrane damage is a more plausible hypothesis. It should be checked by membrane lipoperoxidation assay.

Moreover, endoplasmic reticulum stress, as a modulatory mechanism, triggers two types of cell death based on the level of cell stress, including autophagy and apoptosis. When cells face acute and mild ER stress, autophagy is triggered at a higher level than apoptosis, while in prolonged and severe ER stress, apoptosis is a dominant process over autophagy [29–31]. It has been demonstrated that cancer-derived conditioned media induce dysregulation of calcium homeostasis and consequently endoplasmic reticulum stress, which in turn triggers apoptosis [8]. It appears that a higher generation of iROS in conditioned media may be associated with disruption of protein folding in the ER that eventually promotes cell cycle arrest and induces apoptosis through the unfolded protein response [32].

The exact mechanism by which acidic media affect cell viability remains unclear. Jolly et al. [33] revealed that acidic stress is associated with apoptosis of esophageal cell lines. Therefore, acidic pH may mediate cell cytotoxicity by inducing the apoptosis of NIH/3T3 cells. To support this hypothesis, apoptosis was studied using Bcl2 family proteins, including Bax, Bcl2, Bax/Bcl2 ratio and Annexin-PI flowcytometry. There was no significant difference in the expression of Bcl2 or the Bax/Bcl2 ratio between the different groups, while the level of Bax, an apoptotic protein, was lower in the acidic medium than in the normal medium. Early apoptosis data also showed that although the level of early apoptosis was high in conditioned plus acidic media at 48 h, it was dramatically decreased to less than all groups and instead conditioned media induced a significantly higher level of early apoptosis in cells compared to others. These data are in good agreement with intracellular ROS data. Based on the ER stress mechanism observed in Annexin-PI flowcytometry, the amount of apoptosis in cells treated with acidic media was drastically decreased but significantly less than that in cells treated with conditioned media. Furthermore, the addition of acidic media to conditioned media causes high stress in cells, and cells undergo apoptosis, while over time, some modulatory mechanisms are activated in cells to enhance cell viability. It seems that an acidic environment minimizes the detrimental effect of conditioned media on cell viability by prohibiting ROS generation. On the other hand, in cells treated with conditioned media, prolonged and high-stress conditions, such as intracellular ROS in cells and apoptotic bodies in conditioned media, lead the cell to suicide and apoptosis.

To further study the cell death mechanisms, autophagy was studied using AO staining and LC3-II western blotting. Autophagosome vesicles and the level of LC3-II/LC3-I were drastically increased in acidic media compared to normal media at 48 and 72 h. On the other hand, conditioned media downregulated the LC3-II protein compared to the other groups at 72 h. Moreover, the addition of acidic media to conditioned media shifted cells toward autophagy rather than apoptosis. These results suggest that the acidic stress-mediated induction of autophagy is associated with LC3-II upregulation, but conditioned media likely causes cell apoptosis in addition to the autophagy pathway.

DNA hypomethylation is a feature of cancer cells in the onset and progression of cancer and induces DNA recombination in cells as a dynamic process leading to alterations in expression patterns [34]. Even though, DNA hypomethylation may be considered a marker of tumor diagnosis and progression and tumor grade [34–36]. It is also a target of chemotherapeutic agents [37]. Colocalized DNA methyltransferases (DNMTs), DNA methylation enzymes, and death domain-associated protein (DAXX) are near autophagy-related (ATG) proteins [38]. Decreasing the level of autophagy-related proteins such as ATG4A decreases autophagy in cancer cells, resulting in tumor aggressiveness [39, 40]. The analysis of global DNA methylation using the GC/MS method revealed that DNA methylation in acidic media was significantly lower than in cells in normal media. Not only does acidic media rescue cells treated with conditioned media, but it also decreases DNA hypomethylation in the combination group. On the other hand, the addition of acidic media decreases DNA hypomethylation in acidic plus conditioned media. However, it should be checked whether the DNA methylation content of conditioned media is higher or less than that of acidic media. Recent evidence has suggested that the acidic media of cancer cells may induce epigenetic modifications in cells and genomic instability [34, 41]. Thus, in the present study, it seems that acidic stress may in part promote cell apoptosis by inducing epigenetic modifications. Therefore, these data show that hypomethylation of DNA is likely another mechanism of the acidic media on cell viability. To sum things up, DNA hypomethylation in conditioned plus acidic media decreased compared to that in acidic media, showing another reason to enhance cell viability and rescue from acidic media.

## 5. Conclusion

This study is the first to investigate the separate and combined effects of acidic and conditioned media on cell death mechanisms and DNA hypomethylation in normal cells. The interaction between these two media is important since cancer cells produce a combination of acidic and conditioned media that promote cancer cell signaling. Results indicated that when cancer progresses, normal cells around the cancer cells experience acute stress conditioned through acidic media as a byproduct of cancer cells and exosomes, apoptotic bodies and other biomolecules secreted by cancer cells. This initial intense stress leads cells to commit suicide. Over time, normal cells become accustomed to the condition, and mediators are created to increase cell survival and transform healthy cells into cells with unusual changes. The normal cells under transition changes decide not to commit suicide and instead choose other paths to life. How much more dangerous this scenario is than zombies becoming cells. Cancer cells can affect healthy cells even remotely and by secreting zombies’ biomolecules. In a group of healthy cells that are in this condition, it makes the cells think of suicide, and in the remaining group, it changes to look like a zombie cancer cell. This scenario continues to be overcome by inhibitors and chemotherapy. To design more effective drugs, it is important to know which parts of the secreted material play a stronger role in becoming zombies. To date, most mechanisms have been used to inhibit cell suicide or apoptosis. It is time to consider cell survival mechanisms in affected normal cells adjacent to cancer cells more than cell suicide mechanisms. Based on our findings, it seems that acidic media rescue the cell death potential of conditioned media.

## Supporting information

S1 Data (XLSX)
